# Peptidomimetics Based on *C*-Terminus of Blm10 Stimulate Human 20S Proteasome Activity and Promote Degradation of Proteins

**DOI:** 10.3390/biom12060777

**Published:** 2022-06-02

**Authors:** Katarzyna Cekała, Karolina Trepczyk, Daria Sowik, Przemysław Karpowicz, Artur Giełdoń, Julia Witkowska, Małgorzata Giżyńska, Elżbieta Jankowska, Ewa Wieczerzak

**Affiliations:** Faculty of Chemistry, University of Gdańsk, Wita Stwosza 63, 80-308 Gdańsk, Poland; katarzyna.jedrzejewska@phdstud.ug.edu.pl (K.C.); karolina.trepczyk@phdstud.ug.edu.pl (K.T.); daria.sowik@phdstud.ug.edu.pl (D.S.); przemyslaw.karpowicz@ug.edu.pl (P.K.); artur.gieldon@ug.edu.pl (A.G.); julia.witkowska@ug.edu.pl (J.W.); malgorzata.gizynska@ug.edu.pl (M.G.)

**Keywords:** proteasome, activation, 20S, neurodegenerative diseases, microscale thermophoresis

## Abstract

Degradation of misfolded, redundant and oxidatively damaged proteins constitutes one of the cellular processes which are influenced by the 20S proteasome. However, its activity is generally thought to decrease with age which leads to the gradual accumulation of abnormal proteins in cells and their subsequent aggregation. Therefore, increasing proteasomal degradation constitutes a promising strategy to delay the onset of various age-related diseases, including neurodegenerative disorders. In this study we designed and obtained a series of peptidomimetic stimulators of 20S comprising in their sequences the *C*-terminal fragment of Blm10 activator. Some of the compounds were capable of enhancing the degradation of natively unfolded and oxidatively damaged proteins, such as α-synuclein and enolase, whose applicability as proteasome substrates was evaluated by microscale thermophoresis (MST). Furthermore, they increased the ChT-L activity of the proteasome in HEK293T cell extracts. Our studies indicate that the 20S proteasome-mediated protein substrates hydrolysis may be selectively increased by peptide-based stimulators acting in an allosteric manner. These compounds, after further optimization, may have the potential to counteract proteasome impairment in patients suffering from age-related diseases.

## 1. Introduction

The proteasome is a multienzymatic complex that maintains protein homeostasis and degrades misfolded, redundant, and damaged proteins, which are the precursors of toxic oligomeric forms [1,2]. It exists in equilibrium between 26S and 20S forms. The 20S core is composed of four heteroheptameric rings, stacked and arranged in αββα fashion. Each of the two inner β-rings contains three catalytic subunits (β1, β2, and β5) that exhibit three different substrate cleavage preferences: caspase-like post acidic (C-L, β1/1′), trypsin-like post basic (T-L, β2/2′), and chymotrypsin-like post hydrophobic (ChT-L, β5/5′). The *N*-terminal residues of α-subunits, which point inward to the center of the ring, serve as a gate and restrict access of substrates to the catalytic chamber. In mammalian cells, the 20S core particle can be bound to one or two 19S regulatory subunits (also called PA700), forming so-called 26S proteasome. The role of the 19S is to recognize polyubiquitynylated proteins, unfold them and translocate to the 20S core for degradation in an ATP-dependent manner. In addition to regulation by 19S, 20S can be activated by other regulatory particles [3]. For instance, the proteasome’s gated channel can be opened by the attachment of an 11S activator (also called PA28), which can form either a heteroheptameric ring of alternating PA28α and PA28β subunits or a homoheptameric ring consisting solely of PA28γ. The 20S proteasome can also be activated by a monomeric protein Blm10/PA200 (*Saccharomyces cerevisiae*/human) [4]. Both 11S and Blm10 activate the proteasome in an ATP- and ubiquitin-independent processes, acting in an allosteric manner. Their binding outside the catalytic chamber causes not only unlocking of the entrance gate but also initiates transduction of allosteric signals to the proteolytic sites thus affecting their performance [5,6].

During aging, the processes of oxidation, glycoxidation and other protein modifications intensify. Since they are also accompanied by a progressive decline in the efficiency of the proteasome, observed in several mammalian tissues and cells [7], this leads to the accumulation of abnormal proteins in cells. The impairment of proteasome function leads to cataract and lipofuscin pigments formation, and more importantly to the pathogenesis of severe neurological disorders, such as amyotrophic lateral sclerosis (ALS), Alzheimer’s, Parkinson’s, and Huntington’s diseases [8]. Hence, increasing the proteasomal degradation in a controlled manner appears attractive as a potential therapeutic method [9]. In the last several years the effectiveness of this approach has been confirmed in cellular and animal models. Although the enhancement of proteasome activity has many therapeutic potentials, it is still a relatively poorly explored field [10]. A rational design of allosteric modulators is hampered mostly because of the structural complexity of the enzyme and by limited knowledge about the allosteric mechanism of proteasome activation. Relatively few allosteric small molecule stimulators of the 20S proteasome have been described so far. These are, among others, betulinic acid [11], chlorpromazine [12], dihydroquinazolines [13], and fluspirilene analogs [14].

Another class of proteasome activators are peptides and peptide-based compounds. The example of the peptidic regulator of the proteasome activity is PAP1 peptide [15] which activates the proteasome and is able to prevent protein aggregation in a cellular model of amyotrophic lateral sclerosis. Synthetic allosteric peptide modulators can also be derived from the binding regions of the natural proteinaceous proteasome regulators, 19S and Blm10/PA200. These activators contain a conserved *C*-terminal hydrophobic-tyrosine-any amino acid (HbYX) motifs that in an allosteric manner trigger opening of the 20S gate [16]. The HbYX motifs bind into pockets created by neighboring α subunits and by interactions with the conserved proteasome residues induce rotation in the α subunits and displacement of the Pro17 reverse-turn loop that causes gate opening. Short peptides comprising the HbYX motifs of Rpt2 and Rpt5 subunits of the 19S have been described to enhance 20S-mediated peptide and protein degradation in vitro [17,18]. It has also been shown that Rpt5 peptide competes with Aβ oligomers for binding to the 20S, thus preventing proteasome inhibition [19]. Results of our studies also proved the potential of peptidic activators. We have recently reported that introduction of the HbYX motif to the sequence of proline- and arginine-rich (PR) peptides known as proteasome inhibitors led to conversion of these compounds into strong 20S activators [20]. The PR activators stimulated 20S proteasome in vitro to efficiently degrade model protein substrates and activated proteasome in cultured fibroblasts. Moreover, Blm-pep, a 14-residue peptide which sequence was derived from the Blm10 activator, efficiently stimulated all three activities of human 20S proteasome in a dose-dependent manner [21].

In the present study we found that modification of the sequence of Blm-pep led to novel efficient proteasome activators. The obtained peptidomimetics stimulated the ChT-L and T-L peptidases of the 20S proteasome the most effectively. Moreover, they were capable of enhancing the 20S-mediated degradation of natively unfolded and oxidatively damaged protein substrates, including aggregation-prone α-synuclein.

## 2. Materials and Methods

**Synthesis of M-1–M-12 analogs**

Syntheses of all peptidomimetics were carried out on a solid support (Cl-MPA ProTide Resin (LL)), using a Liberty Blue microwave peptide synthesizer (CEM). Potential activators were synthesized according to standard Fmoc (9-fluorenylmethoxycarbonyl) chemistry. The first amino acid was attached to the resin according to the CEM’s protocol, using 1 M *N,N*′-diisopropylethylamine and 0.125 M potassium iodide. 1 M solution of *N,N*′-diisopropylcarbodiimide in dimethylformamide with an antiracemization additive, 0.5 M ethyl cyano(hydroxyimino)acetate (Oxyma Pure), were used as coupling reagents. Purifications of crude peptides were carried out on C12 semipreparative Jupiter Proteo column (21.2 mm × 250 mm, 4 μm, Phenomenex, Torrance, CA, USA) using RP-HPLC (K2001-Knauer) and a linear gradient of acetonitrile in 0.1% aqueous trifluoroacetic acid. The purity of the synthesized compounds was evaluated by analytical RP-HPLC (Varian ProStar 240) using an XB-C18 Aeris Peptide column (4.6 × 150 mm, 3.6 μm, 100 Å, Phenomenex) or Jupiter Proteo C12 column (4.6 × 250 mm, 4 μm, 90 Å, Phenomenex) and a 30 min linear gradient of 5–80% acetonitrile in 0.1% aqueous TFA with detection wavelength λ  =  223 nm. Identification of pure products was assessed by electrospray ionization ion trap time-of-flight liquid chromatography mass spectrometry (ESI IT TOF LCMS) with a C12 Jupiter Proteo column (2 × 150 mm, 4 μm, 90 Å, Phenomenex).

**Proteasome activity assays**

The influence of the M-1–M-12 analogs on the proteasome catalytic activities was tested using human 20S isolated from erythrocytes and used at the final concentration of 0.001 mg/mL (1.4 nM). Proteasome activities were determined by monitoring the rate of hydrolysis of the appropriate fluorogenic substrates (Suc-LLVY-AMC for chymotrypsin-like (ChT-L), Boc-LRR-AMC for trypsin-like (T-L) and Z-LLE-AMC for caspase-like (C-L) activity (Bachem, Bubendorf, Switzerland)). Stock solutions of the substrates and the tested peptidomimetics were prepared in dimethyl sulfoxide (DMSO). The final DMSO concentration was kept constant at 2%. The final concentration of the substrates was 100 µM, and the M-1–M-12 compounds were tested in the range of 0.5 μM to 10 μM. The activity assays were performed in the 96-well plate format in 50 mM Tris-HCl buffer, pH 8.0, using a 100 μL reaction volume. The release of aminomethylcoumarin (AMC) was measured continuously in 2-min intervals for 60 min, at 37 °C, using Tecan Infinite M200 Pro spectrofluorimeter (Tecan Trading AG, Männedorf, Switzerland). The excitation and emission wavelengths were set at 380 nm and 460 nm, respectively. The change in the slope of the linear section of the AMC emission curves was used to calculate the percentage of the substrate hydrolysis in the presence of allosteric activators, in relation to the control that was regarded as 100%. All activity assays were performed in three independent replicates.

**Molecular modeling**

For the modeling process, three crystal structures of the proteasome were used: human constitutive 20S proteasome (PDB ID: 4R3O), yeast 20S proteasome in complex with Blm10 (PDB ID: 4V7O), and yeast 20S proteasome in complex with Blm-pep activator (PDB ID: 5NIF). The 5NIF experimental structure was used as a template for modeling. In this structure the *C*-terminal part of Blm-pep was visible in the binding pocket located between the α5 and α6 subunits of the yeast proteasome. The *C*-terminal sequence of Blm-pep was modeled in the same place for the human 20S, in which these subunits correspond to α5 and α1, respectively. The lowest energy structure was subsequently optimized in the Amber ff14SB force field [22]. The model was analyzed using RasMol program [23]. The electrostatic and hydrophobic surfaces were calculated using APBS, the Adaptive Poisson-Boltzmann Solver [24], and UCSF Chimera software [25].

**Microscale thermophoresis (MST) assay**

*Protein oxidation*

Commercially available stock solutions of native human enolase 2 (Novus Biologicals, Centennial, CO, USA) and α-synuclein (rPeptide, Watkinsville, GA, USA) were diluted with ddH_2_O to 0.5 mg/mL concentration and incubated with 0.5% H_2_O_2_ for 2 h at 30 °C. The oxidation reactions were stopped by the addition of DTT to the final concentration of 12.5 mM, and then extensively dialyzed overnight to either 10 mM Tris buffer pH 7.4 containing 50 mM KCl and 2.5 mM MgSO_4_ (enolase) or 20 mM Tris buffer pH 7.4 containing 100 mM NaCl (synuclein). The oxidized proteins were used in MST and degradation assays. The oxidation state of α-synuclein and enolase was determined by using MALDI TOF/TOF 5800+ mass spectrometer (ABSciex, Framingham, MA, USA) and TripleTOF 6600+ mass spectrometer with OptiFlow^®^ ESI nano-ion source (Sciex), respectively. The MS spectra are available in Supplementary Materials.

*Protein labeling*

Enolase, α-synuclein and their oxidized forms were labeled using Monolith Protein Labeling Kit RED-NHS 2nd Generation Amine Reactive, NT-647 (Nanotemper, München, Germany), according to the manufacturer instructions. The labeling process was carried out in 50 mM HEPES buffer, pH 7.4, in the protein:NT-647 1:2 ratio. The incubation time was 60 min for enolase and 30 min for α-synuclein, at 21 °C. After labeling, the excess of the fluorescent reagent was removed using the columns provided by the manufacturer and 20 mM Tris-HCl buffer, pH 7.2, containing 200 mM NaCl and 10% glycerol. The obtained labeled proteins were concentrated on Amicon 3K spin-columns (Merck, Darmstadt, Germany). Protein concentrations were determined by measuring absorbance at 280 nm, using UV/Vis spectrophotometer (DU^®^730 Beckman Coulter).

*Protein-proteasome interactions*

Direct interactions between the fluorescently labeled, native and oxidized, proteins and the 20S proteasome were examined using microscale thermophoresis (NanoTemper Monolith NT.115). The labeled protein concentration was kept constant at 250 nM for native and oxidized α-synuclein and 125 nM and 25 nM for native and oxidized enolase, respectively. The labeled proteins were mixed with 16 different concentrations of h20S, prepared as serial dilutions, ranging from 1965 to 0.06 nM and incubated for 60 min (enolase) or 30 min (α-synuclein) in the dark, at 22 °C, in a glass capillary (Monolith NT Premium Capillaries, NanoTemper Technologies GmbH, München, Germany). 20 mM Tris-HCl buffer, pH 7.2, containing 10% glycerol, 0.5 mM DTT, 0.5 mM NaN_3_, 0.125 mM EDTA and 100 mM NaCl was used as an assay buffer. Thermophoresis experiments were performed with IR Laser of 20% radiation intensity for native enolase and native and oxidized α-synuclein, and of 40% intensity for oxidized enolase. The MST curves were fitted with the Hill method, using OriginPro 2021. The EC50 values were calculated based on results obtained in three independent experiments.

**Protein substrate degradation assay**

Enolase and its oxidized form (20 pmol) were dissolved in 20 mM Tris pH 7.4. Native and oxidized α-synuclein (340 pmol) were dissolved in 20 mM HEPES pH 7.4. Human 20S proteasome was pre-activated with 0.01% SDS, which is required in in vitro studies to loosen the enzyme compact structure. The samples were prepared by mixing h20S, the protein and either DMSO (control) or the modulator dissolved in DMSO. Total sample volume was 10 µL. Final concentration of the organic solvent never exceeded 0.05%. The 20S:protein ratio was 1:20 and 1:340 for enolase and α-synuclein, respectively. The samples were incubated at 37 °C for 3 h, only in the case of native α-synuclein, due to its higher degradation rate, this time was shortened to 1 h (Figure S3). The reaction was stopped with 4 × Laemmli buffer, then heated at 75 °C for 10 min. In the case of α-synuclein, due to the higher content of the protein in the incubated samples, this step was preceded by diluting the reaction mixture with 25 µL of the assay buffer. The results were analyzed electrophoretically after loading 8 µL of each sample onto a 10% (for enolase) or 12% (for synuclein) SDS–PAGE gel.

The amounts of undigested proteins were calculated from gels stained with Coomassie Blue-based reagent, InstantBlue^TM^ (Figures S4 and S5). The quantitative image analysis was carried out with Quantity One^®^ 1-D analysis software (Bio-Rad, Hercules, CA, USA). The band intensities of a protein incubated with a modulator and/or h20S were compared with the intensity of the band corresponding to the intact protein, considered to be 100%. Each value represents an average of data from at least three experiments and is presented as mean ± SEM. Statistical analysis was performed with SigmaPlot 14.0. Differences between experimental groups were analyzed using one-way ANOVA followed by Bonferroni post hoc test for pairwise comparison. A value of *p* < 0.05 was considered statistically significant.

**Proteasome activity in HEK293T cell lysates**

Human embryonic kidney cells (HEK293T) were maintained in Dulbecco’s Modified Eagle Medium (DMEM) supplemented with 10% Fetal Bovine Serum and penicillin/streptomycin (100 units/mL/100 µg/mL), at 37 °C with 5% CO_2_. Cells were grown in a T-75 flask to ~80% confluency and then were lysed using Reporter Lysis Buffer (Promega, Madison, WI, USA) and the manufacturer’s protocol. Protein content was measured using BCA assay. Stock solutions of the fluorogenic substrate (Suc-LLVY-AMC) and the tested peptidomimetics were prepared in dimethyl sulfoxide (DMSO). Potential activators were tested in the following concentrations: 1, 5, 10, 25 and 50 μM. The final DMSO concentration was kept constant at 2%. The activity assays were performed analogously to those described in the proteasome activity assays subsection, in the 96-well plate format in 50 mM Tris-HCl buffer, pH 8.0, using a 100 μL reaction volume. The final concentration of the fluorogenic substrate and cell lysate proteins were: 100 μM and 20 µg/mL, respectively. As a negative control, 1 μM bortezomib solution was used.

**MTT assay**

The cytotoxicity of tested compounds was estimated using the MTT assay. HEK293T cells were seeded in 96-well plates (7 × 10^3^ cells per well) and were incubated for 2 days at 37 °C with 5% CO_2_. Next, growth medium was substituted with medium supplemented with indicated concentrations of tested compounds. Cells were incubated for 24 h, then a 10× concentrated MTT (Acros Organics, Geel, Belgium) solution in PBS buffer was added to a final concentration of 0.5 mg/mL. After 4 h incubation at 37 °C, purple formazan, produced as a result of MTT reduction by metabolically active cells was quantified by measuring the absorbance at 570 nm with a reference filter of 690 nm.

## 3. Results and Discussion

### 3.1. Design of the Peptidomimetics

The sequences of M-1–M-12 analogs were designed based on our Blm-pep activator, which in turn was derived from the sequence of natural proteasome activator, Blm10 protein. In crystal structures of the complexes of Blm-pep and Blm10 with yeast proteasome (PDB ID: 5NIF and 4V7O, respectively) the *C*-terminus of the activator (LWRSYYA) is located between the α5 and α6 subunits. According to the published data, interactions of Blm10 protein with the residues of the α5 subunit are necessary for the enzyme activation [4]. Therefore, we decided to analyze this area in detail. The starting point was modeling of Blm-pep in the same α5/α6 pocket of the human 20S proteasome, in which these subunits correspond to α5 and α1, respectively.

**Figure 1:**
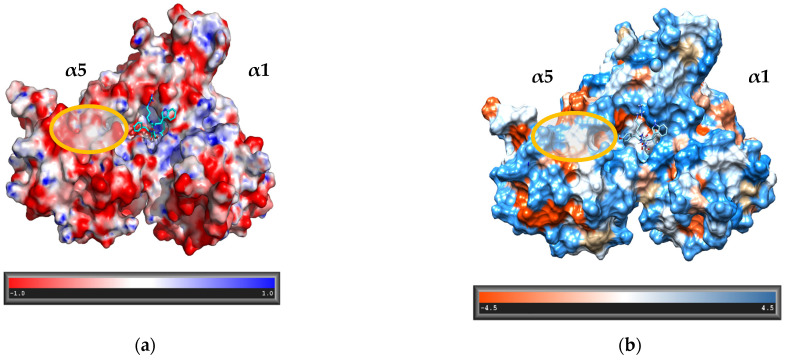
A fragment of the alpha torus of the proteasome. The visible part of the ligand is located between α5 and α1 subunits (corresponding to the α5 and α6, respectively, in yeast 20S) (**a**) APBS-generated electrostatic surface; (**b**) hydrophobic surface (amino acid hydrophobicity is presented in the Kyte-Doolittle scale [26]).

The calculation of electrostatic and hydrophobic surfaces (Figure 1) allowed us to propose a set of potential activators that would align on the α5 side of the pocket occupied by their *C*-terminal part and extend along this surface to a distance of about 10 Å. In the modeled structures the *N*-terminal part of the polypeptide chain was connected with the *C*-terminal binding region through either two, three or four Gly residues. The capacity of each linker to enable interactions of the *N*-terminal region with the α5′s hydrophilic areas was then evaluated through molecular dynamics simulations. The most promising results were obtained for the longest linker, and this information was utilized in designing M-1–M-12 peptidomimetics (Table 1). During the synthesis, the 4Gly-linker was replaced by diethylene glycol moiety (Peg2). We also decided to introduce at the *C*-terminal position of our analogs a serine residue in place of naturally occurring alanine to allow the formation of a hydrogen bond with Gly76 of the α1 subunit, thus strengthening the interactions with the residues at the bottom of the proteasome binding pocket.

### 3.2. Stimulating Capacity of M-1–M-12 Analogs

**Figure 2:**
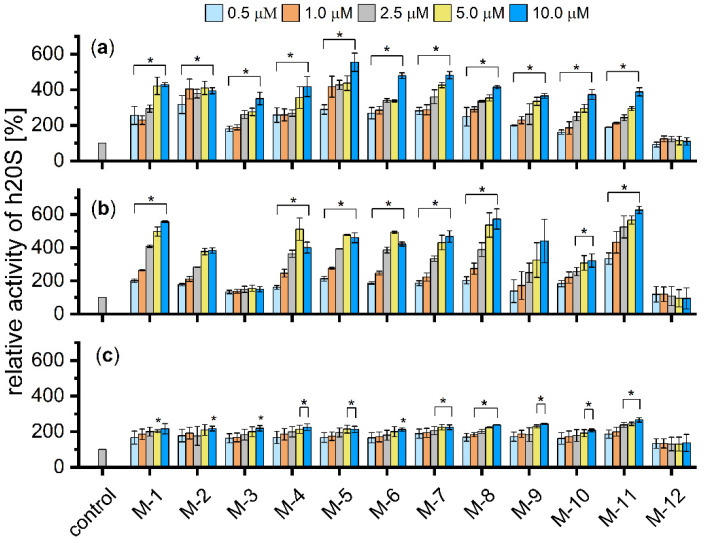
The capacity of M-1–M-12 analogs for stimulating (**a**) ChT-L, (**b**) T-L, (**c**) C-L peptidases of human 20S proteasome. Most of the compounds (except M-12) increased ChT-L and T-L activity of the 20S in a dose-dependent manner. All activity assays were performed in three independent replicates. Results are expressed as a percentage of activity of the 20S alone and are presented as the mean ± SEM. Ordinary one-way ANOVA analysis was used to determine statistical significance (* *p* < 0.05).

The influence of the synthesized peptidomimetics on the proteolytic activity of the human 20S proteasome was analyzed using small fluorogenic peptide substrates containing a 7-amino-4-methylcoumarin reporter group (AMC). The activity assays demonstrated that most of the compounds were effective at stimulating the ChT-L peptidase (Figure 2). At 5 μM each analog, except M-12 caused 2.7–4.4 fold increase in the ChT-L activity, with the strongest stimulation induced by compounds M-5 and M-7. These compounds contain a basic amino acid residue at position 1 (homoarginine and lysine, respectively), and aspartic acid at position 6, in place of leucine naturally occurring at this position in Blm10. The efficient stimulation of h20S caused by these two compounds corroborates the results obtained by molecular dynamics simulation, which indicated that Asp at position 6 could create a salt bridge with an arginine and/or lysine residues of the 20S subunits, thus strengthening the protein–peptidomimetic interaction. Remarkably, the compound M-12, in which the same profitable Leu→Asp exchange was accompanied by the substitution of tyrosine residue at position 10 by its *D* isomer, did not show stimulation capacity for neither ChT-L nor T-L peptidases. This indicates the importance of maintaining the appropriate stereochemistry of residues forming the conserved *C*-terminal hydrophobic-tyrosine-any amino acid (HbYX) motif that triggers the proteasome gate opening [16].

The obtained analogs also enhanced the T-L activity of proteasome. Interestingly, a weaker effect was observed for the compound M-3, which differed only by Asn residue at position 4 from the analog M-11, which displayed the strongest ability to activate this peptidase. Simultaneously, both compounds exhibited similar activities towards the ChT-L and C-L peptidases, which indicates that the replacement of only one amino acid residue can lead to an allosteric modulator that specifically activates proteasome at one or two, but not all three, catalytic sites.

### 3.3. Microscale Thermophoresis (MST) Results

**Figure 3:**
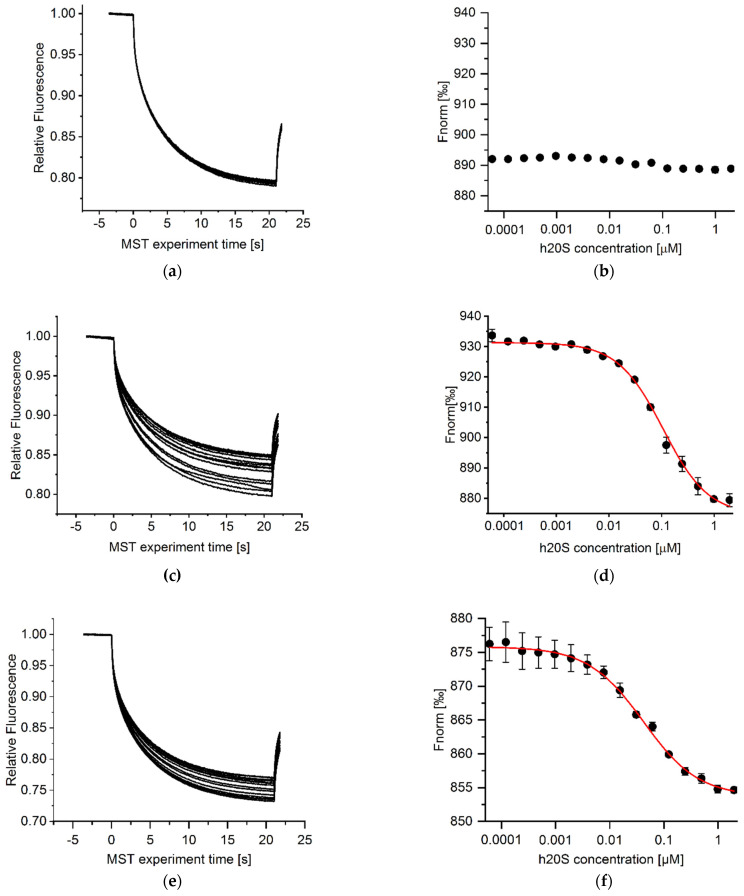

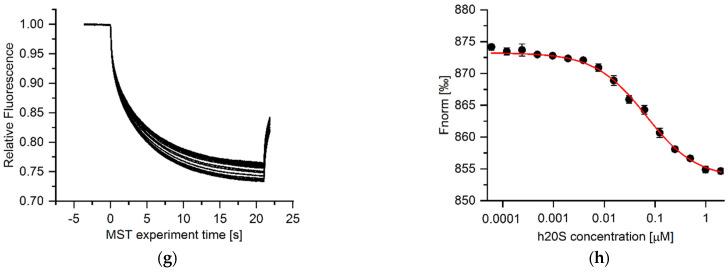
Left panels: MST time traces for the titration of 20S proteasome (from 0.06 to 1965 nM) against (**a**) enolase, (**c**) oxidized enolase, (**e**) α-synuclein, (**g**) oxidized α-synuclein. Representative data of three measurements is shown. Right panels: The normalized fluorescence was plotted against increasing 20S concentration to determine its binding affinity to enolase (**b**), oxidized enolase (**d**), α-synuclein (**f**), oxidized α-synuclein (**h**). MST curves were fitted using Hill equation, and EC50 values were calculated for α-synuclein (32.3 ± 1.3 nM), its oxidized form (67.6 ± 8.9 nM), and oxidized enolase (87.8 ± 12.9 nM) based on results derived from three independent experiments. Native enolase did not bind to 20S proteasome.

Although the AMC containing peptide substrates are convenient and give the general idea about the possibility of stimulating the activity of the proteasome by the tested compounds, they do not mimic the endogenous substrates of the 20S. To further study the influence of our analogs on the protein degradation we selected two proteins that varied in length/size and the tertiary structure, namely human enolase and α-synuclein. The two native proteins were studied along with their oxidized forms, since oxidatively damaged proteins are known as the main substrates of the 20S core particle [27]. The 434-residue thermolabile enolase is a protein with defined tertiary structure whereas α-synuclein is an intrinsically disordered protein whose aggregates are involved in Parkinson’s disease development [28,29]. To determine applicability of the model proteins as proteasome substrates, we conducted MST experiments. The measurements were made on NT647-labeled model proteins in the presence of unlabeled proteasome. The thermophoretic mobility of fluorescently marked proteins was recorded at different proteasome concentrations, and these data were used to calculate binding affinities. With increasing proteasome concentration, a decrease in the MST signal intensity was observed for α-synuclein, oxidized synuclein and oxidized enolase, which indicated a growing number of molecules binding to the enzyme (Figure 3c–h). The binding curves were fitted with the Hill method which allowed to obtain EC50 values. Comparison of these values for α-synuclein (EC50 32.3 ± 1.3 nM), its oxidized form (EC50 67.6 ± 8.9 nM), and oxidized enolase (EC50 87.8 ± 12.9 nM) revealed the tightest binding of the native synuclein, whereas its oxidized form as well as the oxidized enolase exhibited 2-fold lower binding affinity to 20S proteasome. In contrast, enolase in its native form did not bind to 20S (Figure 3a,b).

### 3.4. Degradation of Model Proteins

In the next step of our study, we tested the degradation of enolase, α-synuclein, and their oxidized forms by 20S proteasome, in the presence and the absence of M-1–M-11 analogs. M-12 was discarded due to the lack of stimulating capacity exhibited in the activity assays with the AMC-tagged substrates.

**Figure 4:**
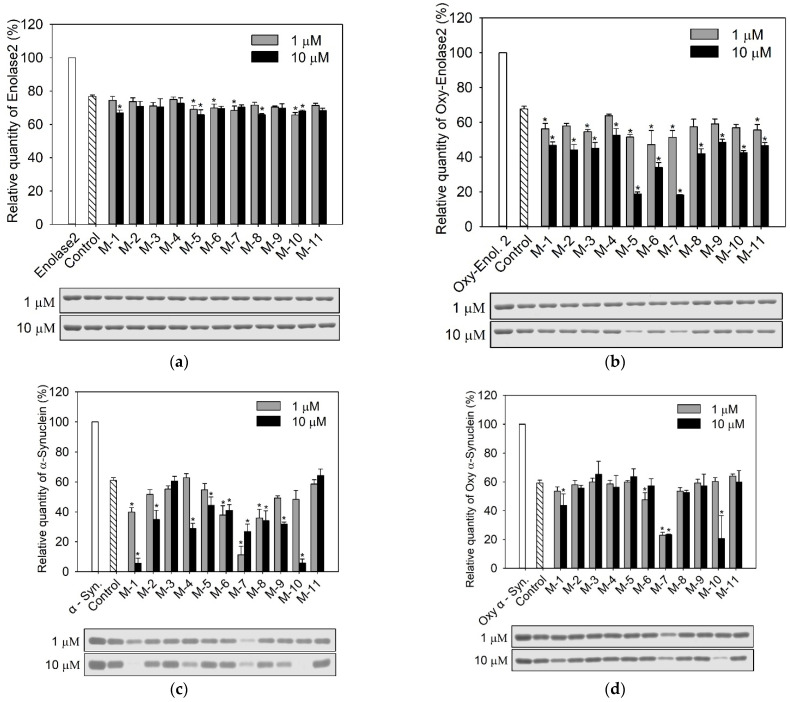
The influence of M-1–M-11 analogs on degradation of protein substrates, (**a**) enolase, (**b**) oxidized enolase, (**c**) α-synuclein, (**d**) oxidized α-synuclein, by the human 20S proteasome. Relative quantities of undigested proteins were determined based on electrophoretic separation of the protein samples after their incubation with the modulators and 20S proteasome or the proteasome alone (control). Representative SDS-PAGE electrophoregrams with Coomassie-stained bands are presented below the charts. At 10 µM, M-5 and M-7 stimulated the degradation of oxidized enolase, whereas none of the tested compounds accelerated the digestion of its native form. Native α-synuclein was almost completely degraded in the presence of M-1 and M-10 at 10 µM concentration. M-7 (10 µM) influenced the most the degradation of oxidized synuclein. The results marked with an asterisk are statistically significant (*p* < 0.05).

Figure 4 shows that the native enolase is degraded by 20S proteasome less efficiently than its oxidized form. Moreover, none of M-1–M-11 analogs was able to enhance the native protein degradation rate. In contrast, some of the analogs were quite efficient in promoting proteasomal degradation of the oxidized enolase. The best stimulators were compounds M-5 and M-7. Stimulation with 10 μM concentration of these compounds led to more than 3-fold acceleration of oxidized enolase digestion. The obtained results demonstrate that the compounds M-5 and M-7 which caused the biggest increase of the proteasome activity in the assays with AMC-substrates, accelerated the most the degradation of this oxidized protein.

In contrast to enolase, the modulators did not significantly enhance the degradation of the oxidized form of α-synuclein, except the compound M-7, which at both 1 and 10 μM concentration increased the level of oxidized protein hydrolysis about 3-fold. Analog M-7 accelerated also the degradation of native α-synuclein, but surprisingly the effect was greater at its lower concentration. It suggests that the compound may share the binding site with α-synuclein and therefore its higher concentration obstructs proteasome activation, which reportedly can be mediated through interactions of exposed hydrophobic regions of the intrinsically disordered proteins. Remarkably, native α-synuclein was almost completely digested in the presence of compounds M-1 and M-10 at their 10 μM concentration. It is quite surprising that these two modulators show such a similar effect since they differ significantly in both their *N*- and *C*-terminal sequences. This shows how difficult it is to rationally design allosteric compounds, especially when the goal of their action is such a huge, multienzymatic complex as the proteasome.

### 3.5. Stimulation of Proteasome Activity in Cell Culture

Encouraged by the observed influence of M-1, M-5, and M-7 peptidomimetics on the model proteins degradation, we investigated if they were capable of enhancing the proteasome activity in HEK293T cells. The M-12 compound was included in the studies as a control since it did not stimulate 20S in in vitro activity assays with the AMC-tagged substrates.

**Figure 5:**
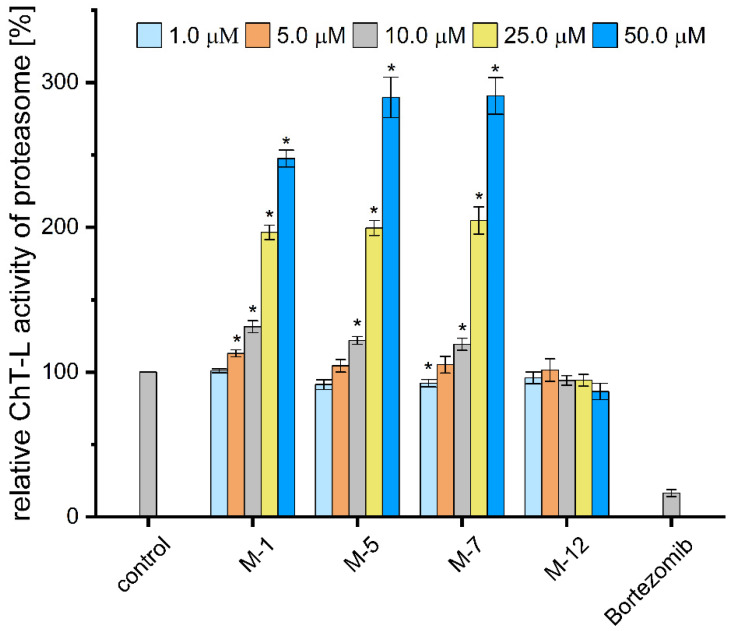
ChT-L peptidase of proteasome was activated up to 3-fold with 50 µM M-5 and M-7 in cell extracts prepared from HEK293T cells. M-12 failed to stimulate the ChT-L peptidase in cell lysates, exactly as with the purified 20S proteasome. The selective proteasome inhibitor, 1 µM bortezomib, used as a negative control, completely blocked the cell capacity of degrading Suc-LLVY-AMC substrate. The results are presented as the mean of three independent replicates ± SEM. Ordinary one-way ANOVA analysis was used to determine statistical significance (* *p* < 0.05).

Total cell lysates were treated with the tested modulators at concentrations ranging from 1 to 50 µM. Figure 5 shows that M-12 had no influence on proteasome activity. In contrast, the ChT-L activity compared to vehicle control increased 2 and 3-fold in cell extracts treated with 25 µM and 50 µM concentrations of M-1, M-5, and M-7 peptidomimetics, respectively. Remarkably, even the highest concentration of M-1, M-5, and M-7 did not exert significant cytotoxic effect on HEK293T cells (Figure S6) which indicates that tested analogs can be promising candidates for further structure-activity studies and development of more drug-like compounds.

## 4. Conclusions

We have designed and synthesized peptidomimetics comprising in their sequences the *C*-terminal fragment of Blm10/Blm-pep activator. Some of the obtained analogs, especially compound M-7, were capable of increasing significantly the proteasome propensity to degrade natively unfolded and oxidatively damaged model proteins, aggregation-prone α-synuclein and human enolase 2. Our results showed that peptide-based stimulators, through transmission of allosteric signals to the α-gate and catalytic sites, may selectively enhance the protein substrates hydrolysis. Furthermore, they increased the ChT-L activity of proteasome in HEK293T cell extracts which indicates that further modification of their sequences rendering them proteolytically stable and cell permeable may yield the proteasome activators capable of diminishing the accumulation of impaired proteins in cells and constitute a promising therapeutic strategy to delay the onset of age-related amyloid depositions and neurodegeneration.

## Figures and Tables

**Table 1:** The sequences of M-1–M-12 analogs.

Compound	Sequence											
	1	2	3	4	5	6	7	8	9	10	11	12
**M-1**	Y	S	Q	E	Peg2	L	W	R	S	Y	Y	S
**M-2**	E	N	S	K	Peg2	L	W	R	S	Y	Y	S
**M-3**	K	N	S	N	Peg2	L	W	R	S	Y	Y	S
**M-4**	N	N	S	E	Peg2	L	W	R	S	Y	Y	S
**M-5**	Har *	N	S	N	Peg2	D	W	R	S	Y	Y	S
**M-6**	K	N	E	N	Peg2	L	W	R	S	Y	Y	S
**M-7**	K	N	S	N	Peg2	D	W	R	S	Y	Y	S
**M-8**	L	N	S	N	Peg2	L	W	R	S	Y	Y	S
**M-9**	E	N	S	N	Peg2	L	W	R	S	Y	Y	S
**M-10**	K	N	S	N	Peg2	D	W	Cit *	S	Y	Y	S
**M-11**	K	N	S	K	Peg2	L	W	R	S	Y	Y	S
**M-12**	K	N	S	N	Peg2	D	W	R	S	dY *	Y	S

* Har—homoarginine, Cit—citrulline, dY—*D*-tyrosine.

## Data Availability

Not applicable.

## Supplementary Materials

The following supporting information can be downloaded at: https://www.mdpi.com/article/10.3390/biom12060777/s1, Supplementary Figures: S1–S6; Supplementary files: Raw data of activity assays; Raw data of MST experiments; Raw data of activity assays in cell lysates.
