# Supplementary material for: Peptidomimetics Based on C-Terminus of Blm10 Stimulate Human 20S Proteasome Activity and Promote Degradation of Proteins
Source: Biomolecules. 2022 Jun 2;12(6):777. doi: 10.3390/biom12060777 (PMC9221443; doi:10.3390/biom12060777)
Supplement: Supplementary file 1 [file biomolecules-12-00777-s001.zip › Supplementary Figures.zip!/Supplementary Figures.pdf]

Supplementary Figures

a.

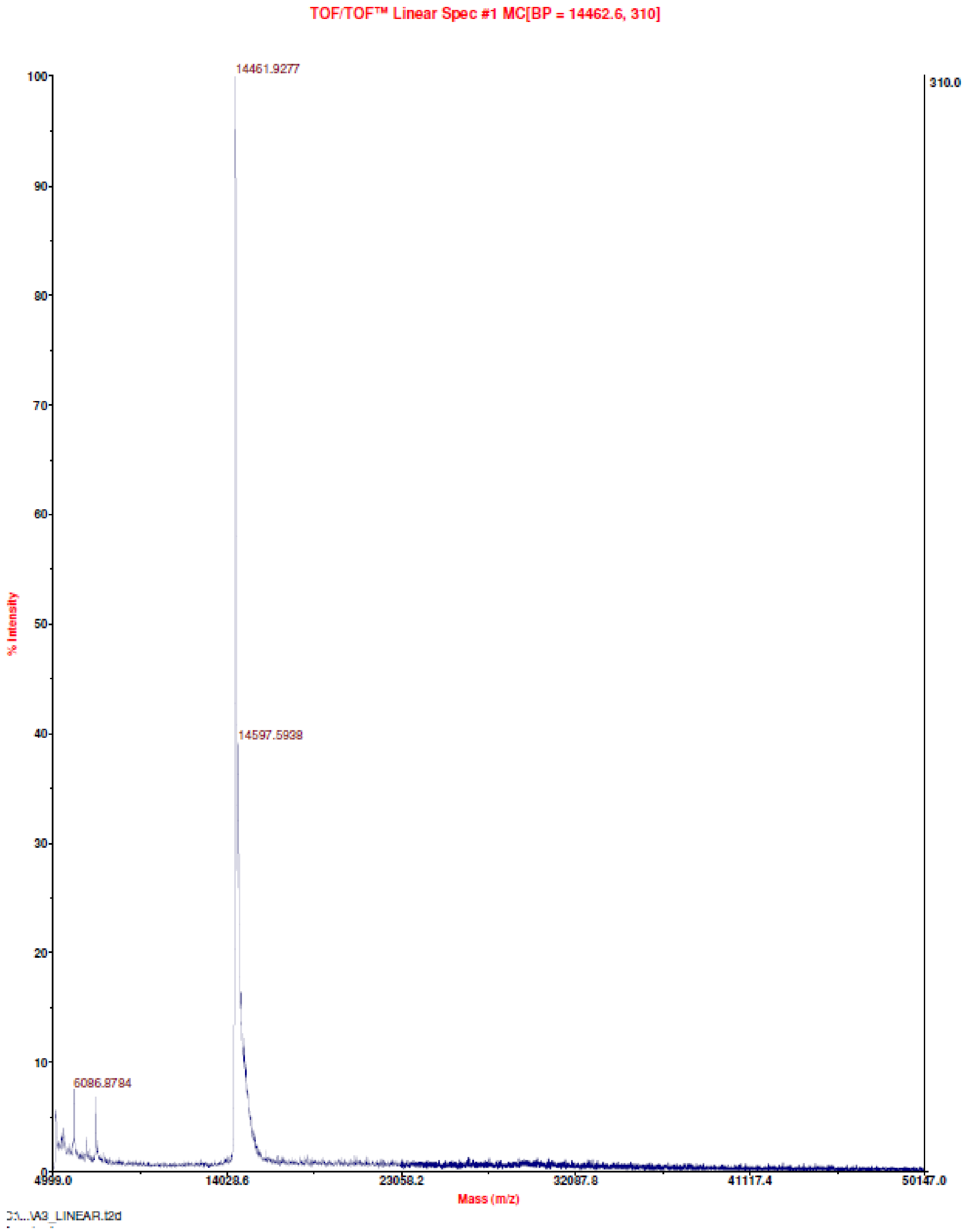

b.

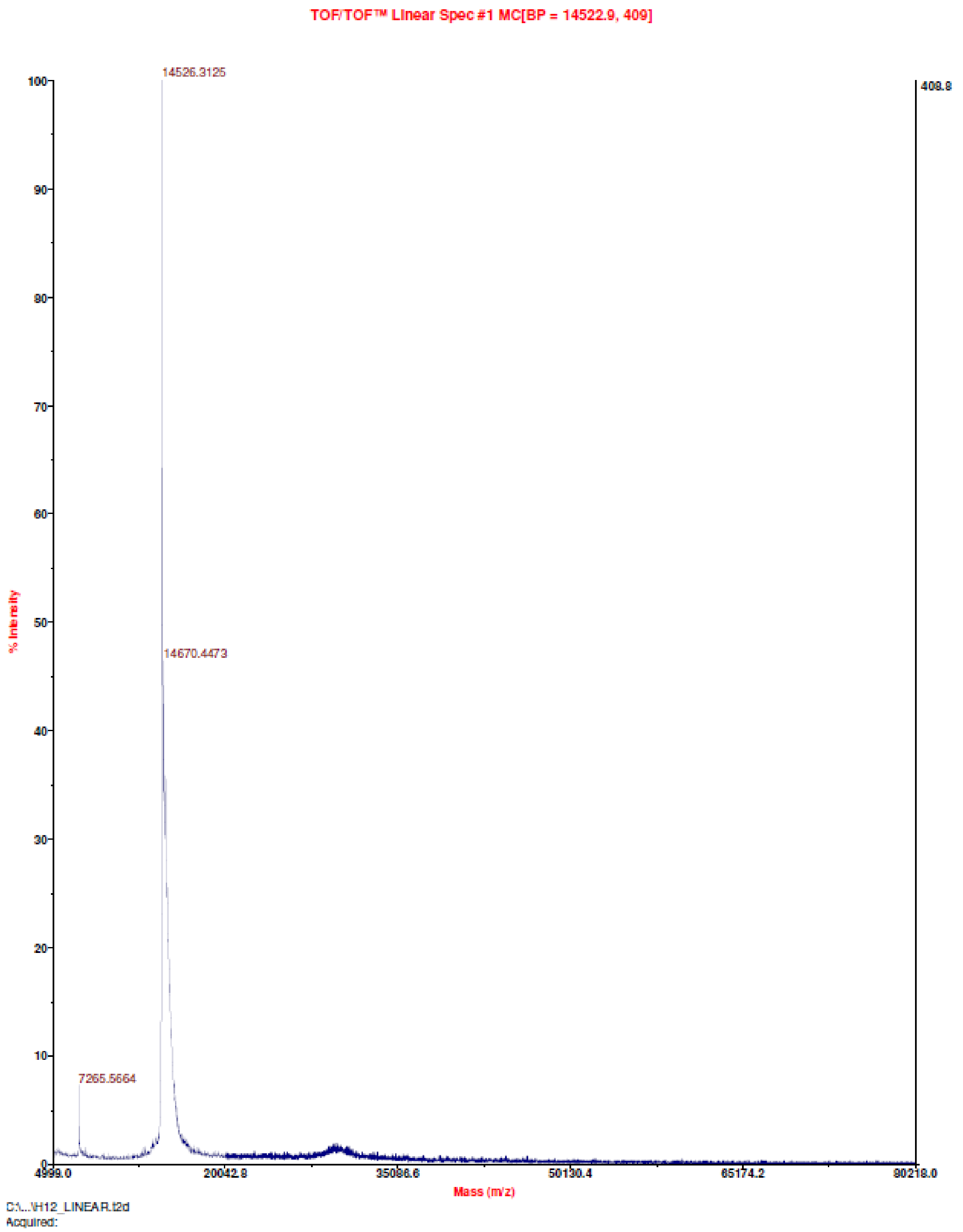

Figure S1. MS spectrum of a) α-synuclein, b) oxidized synuclein. The oxidized form of α-synuclein has a molecular weight of 14.526 kDa. The 64 Da increase in the molecular weight corresponds to four additional oxygen atoms.

a.

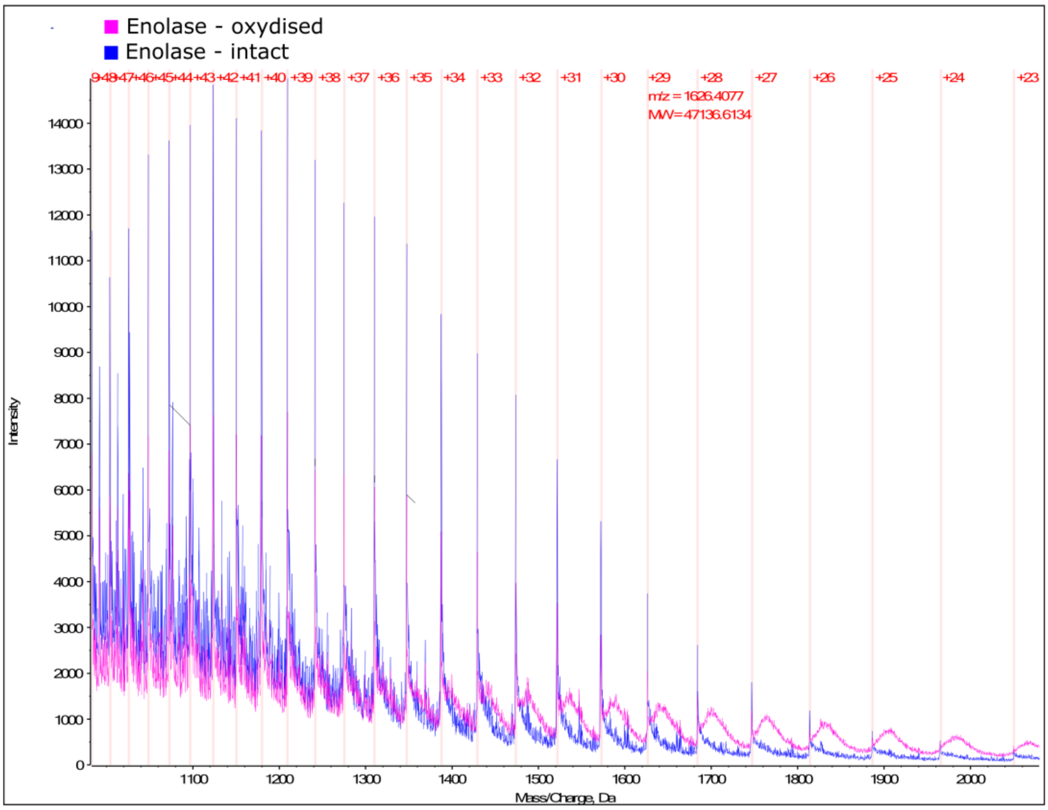

b.

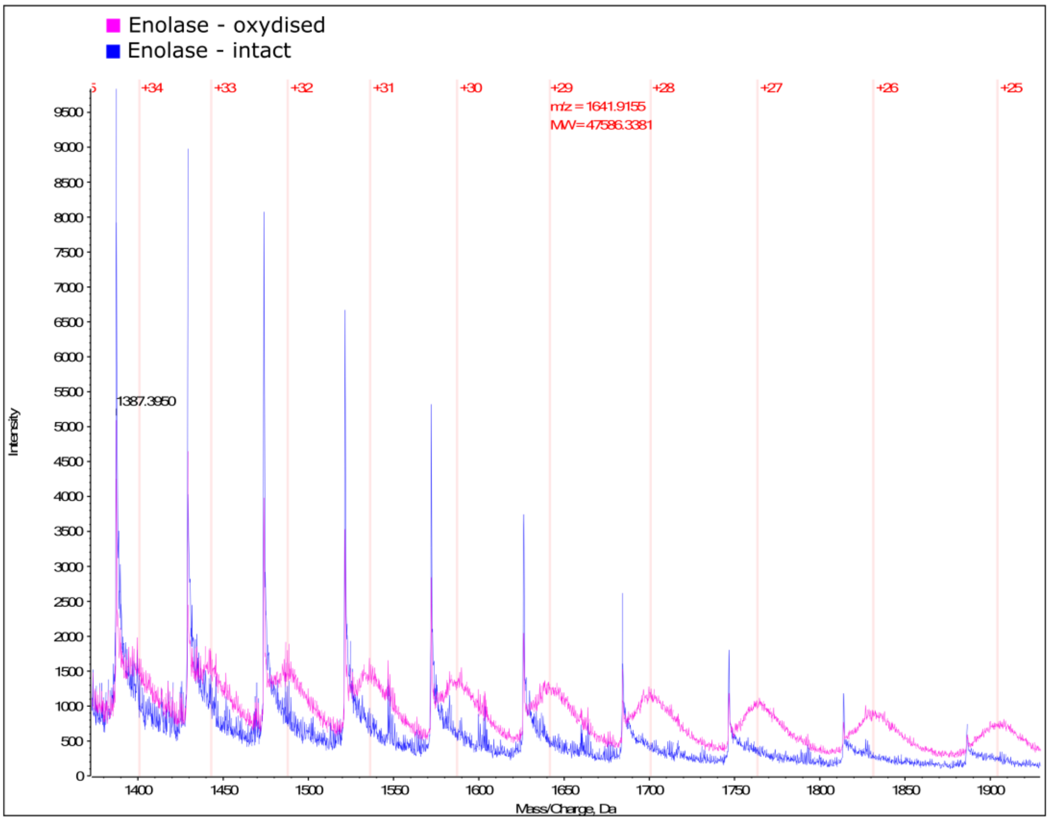

Figure S2. Ion chromatogram of native and oxidized enolase, the distinct ions were marked in red (a, b – enlargement of the fragment of ion chromatogram). The experimentally determined molecular weight of native protein is 47.136 Da, whereas the molecular weight of oxidized enolase is on average 47.586 Da. Treatment of the protein with H_2O_2 led to oxidative damage of enolase which made the determination of the exact number of oxidized residues impossible.

a.
b.

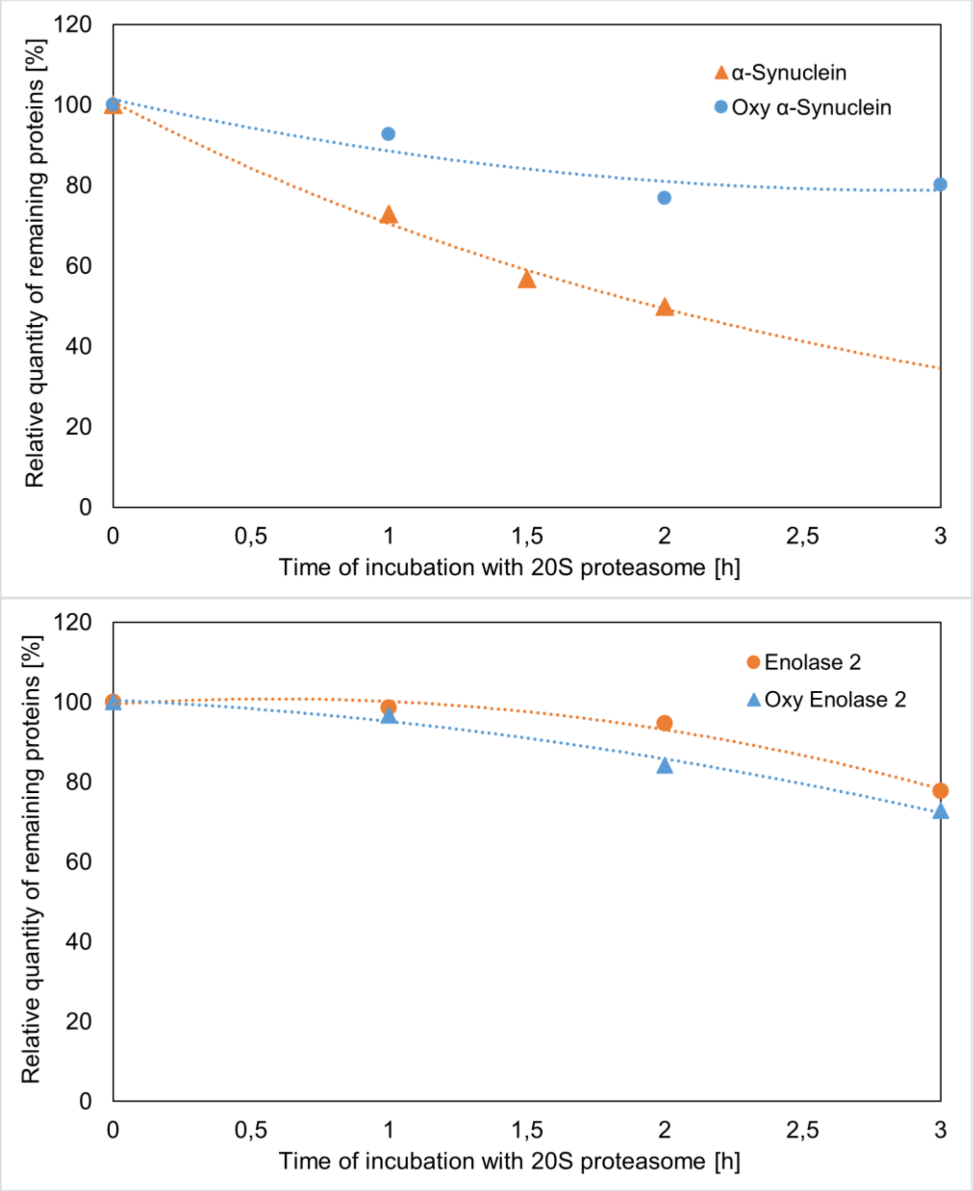

Figure S3. Examples of time-course kinetics of degradation of: (a) native and oxidized α-synuclein; (b) native and oxidized enolase 2. The h20S:protein ratio was 1:340 and 1:20 for synuclein and enolase, respectively. The amounts of undigested proteins were estimated based on SDS PAGE gels stained with Coomassie Blue-based reagent, InstantBlue™, by comparing the intensity of bands corresponding to the proteins incubated with and without h20S. The quantitative image analysis was carried out with Quantity One® 1-D analysis software (Bio-Rad).

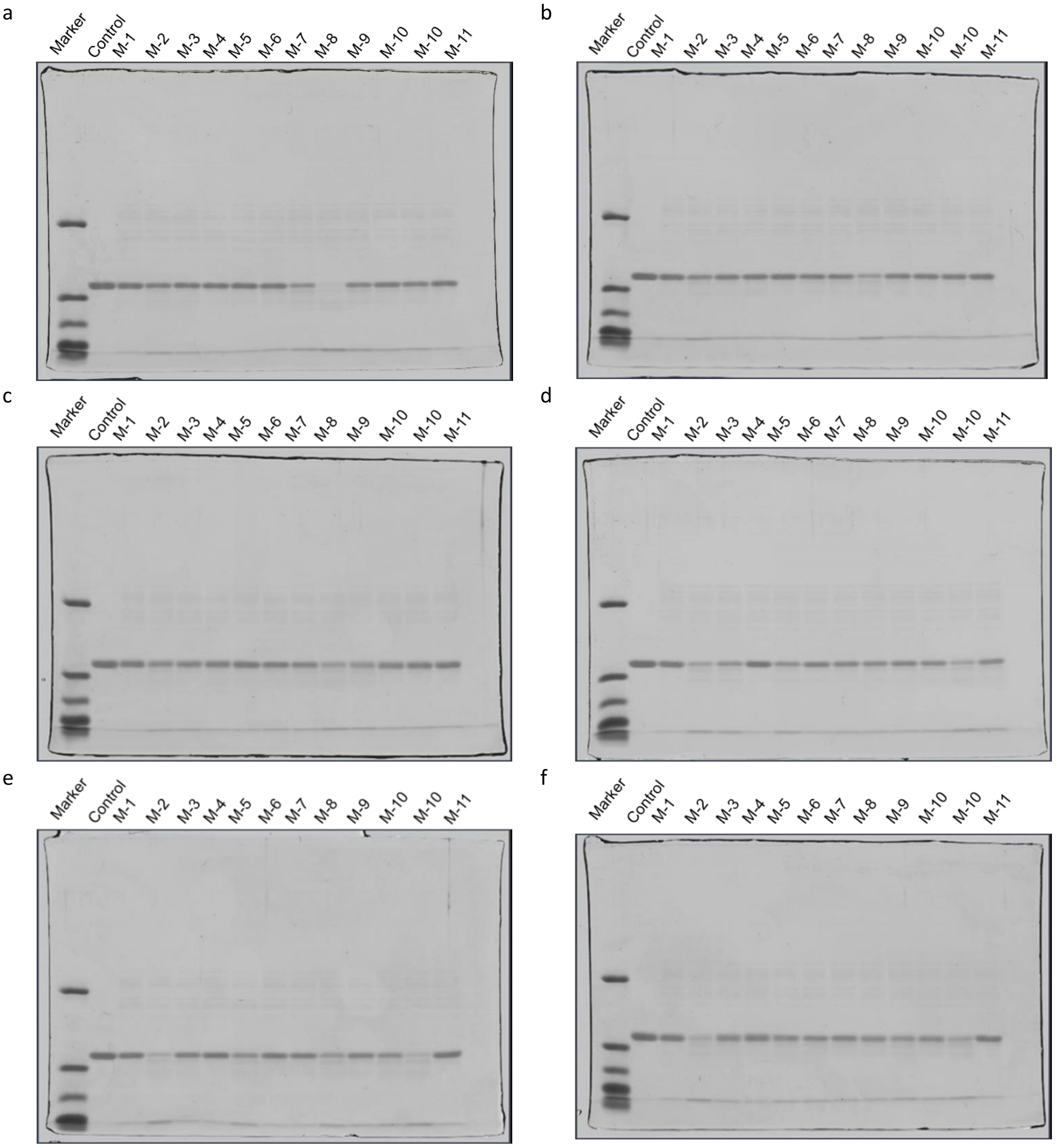
a
Marker
Control
M-1
M-2
M-3
M-4
M-5
M-6
M-7
M-8
M-9
M-10
M-10
M-11
b
Marker
Control
M-1
M-2
M-3
M-4
M-5
M-6
M-7
M-8
M-9
M-10
M-10
M-11
c
Marker
Control
M-1
M-2
M-3
M-4
M-5
M-6
M-7
M-8
M-9
M-10
M-10
M-11
d
Marker
Control
M-1
M-2
M-3
M-4
M-5
M-6
M-7
M-8
M-9
M-10
M-10
M-11
e
Marker
Control
M-1
M-2
M-3
M-4
M-5
M-6
M-7
M-8
M-9
M-10
M-10
M-11
f
Marker
Control
M-1
M-2
M-3
M-4
M-5
M-6
M-7
M-8
M-9
M-10
M-10
M-11

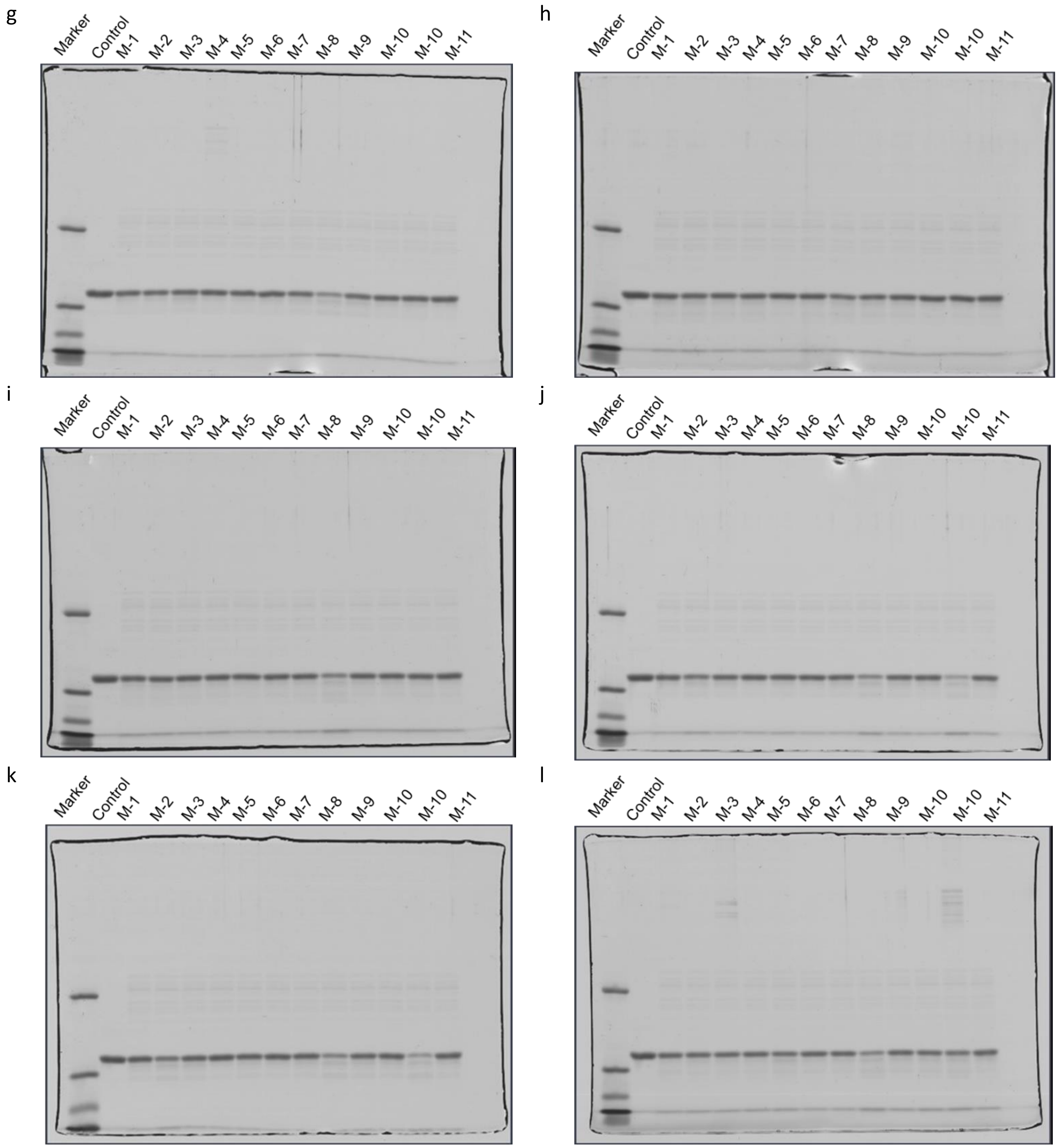

Figure S4. SDS PAGE gels of α-synuclein (a-f), and oxidized synuclein (g-l) degradation by the 20S in the presence of the tested peptidomimetics at 1 µM (a-c, g-i) and 10 µM (d-f, j-l) concentration. The experiments were conducted in three replicates. The unstained polypeptide SDS-PAGE standard (Biorad) was used as marker (bands: 1.4, 3.4, 6.5, 14.4, 16.9, and 26.6 kDa).

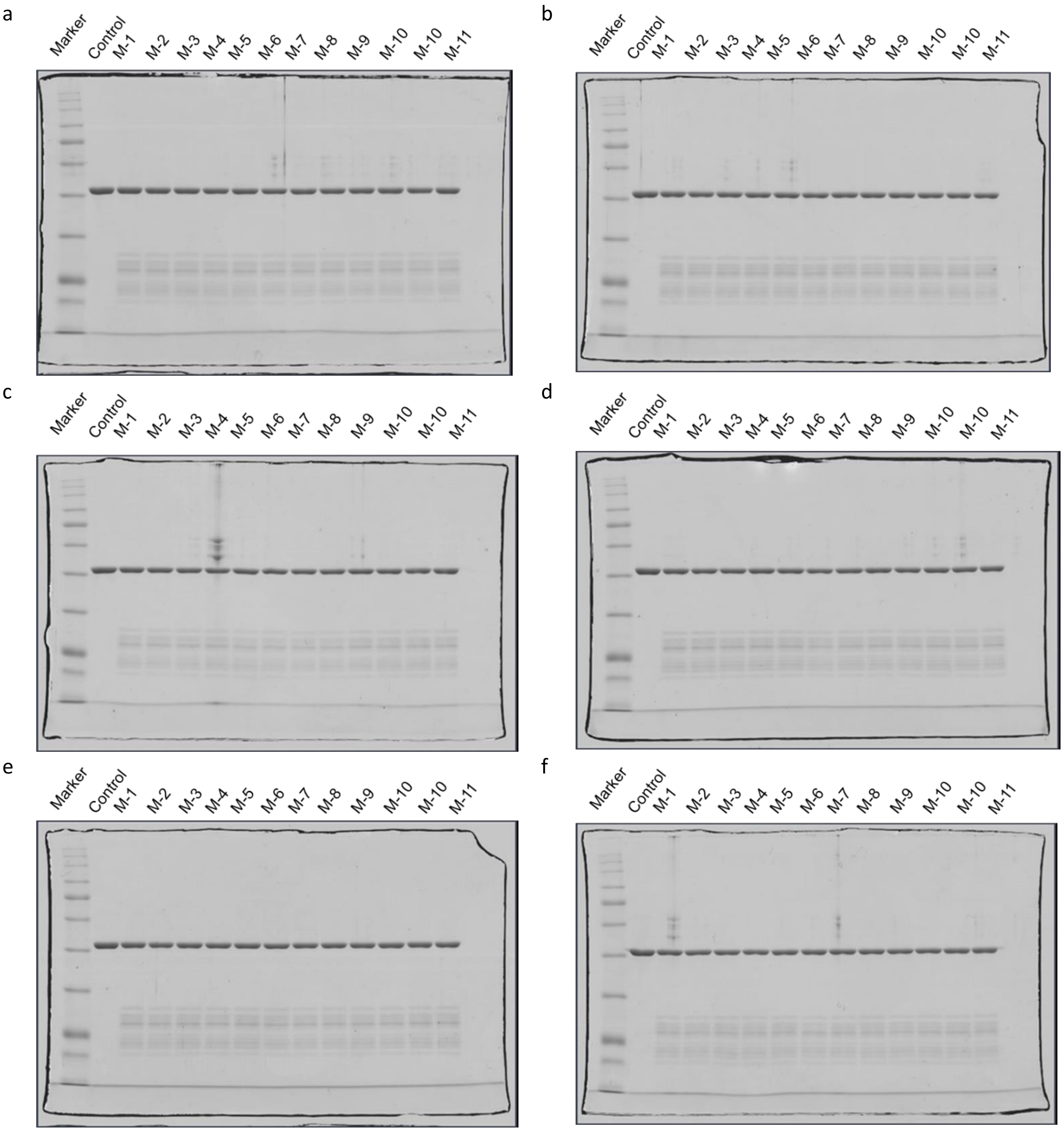
a
Marker Control M-1 M-2 M-3 M-4 M-5 M-6 M-7 M-8 M-9 M-10 M-10 M-11
b
Marker Control M-1 M-2 M-3 M-4 M-5 M-6 M-7 M-8 M-9 M-10 M-10 M-11
c
Marker Control M-1 M-2 M-3 M-4 M-5 M-6 M-7 M-8 M-9 M-10 M-10 M-11
d
Marker Control M-1 M-2 M-3 M-4 M-5 M-6 M-7 M-8 M-9 M-10 M-10 M-11
e
Marker Control M-1 M-2 M-3 M-4 M-5 M-6 M-7 M-8 M-9 M-10 M-10 M-11
f
Marker Control M-1 M-2 M-3 M-4 M-5 M-6 M-7 M-8 M-9 M-10 M-10 M-11

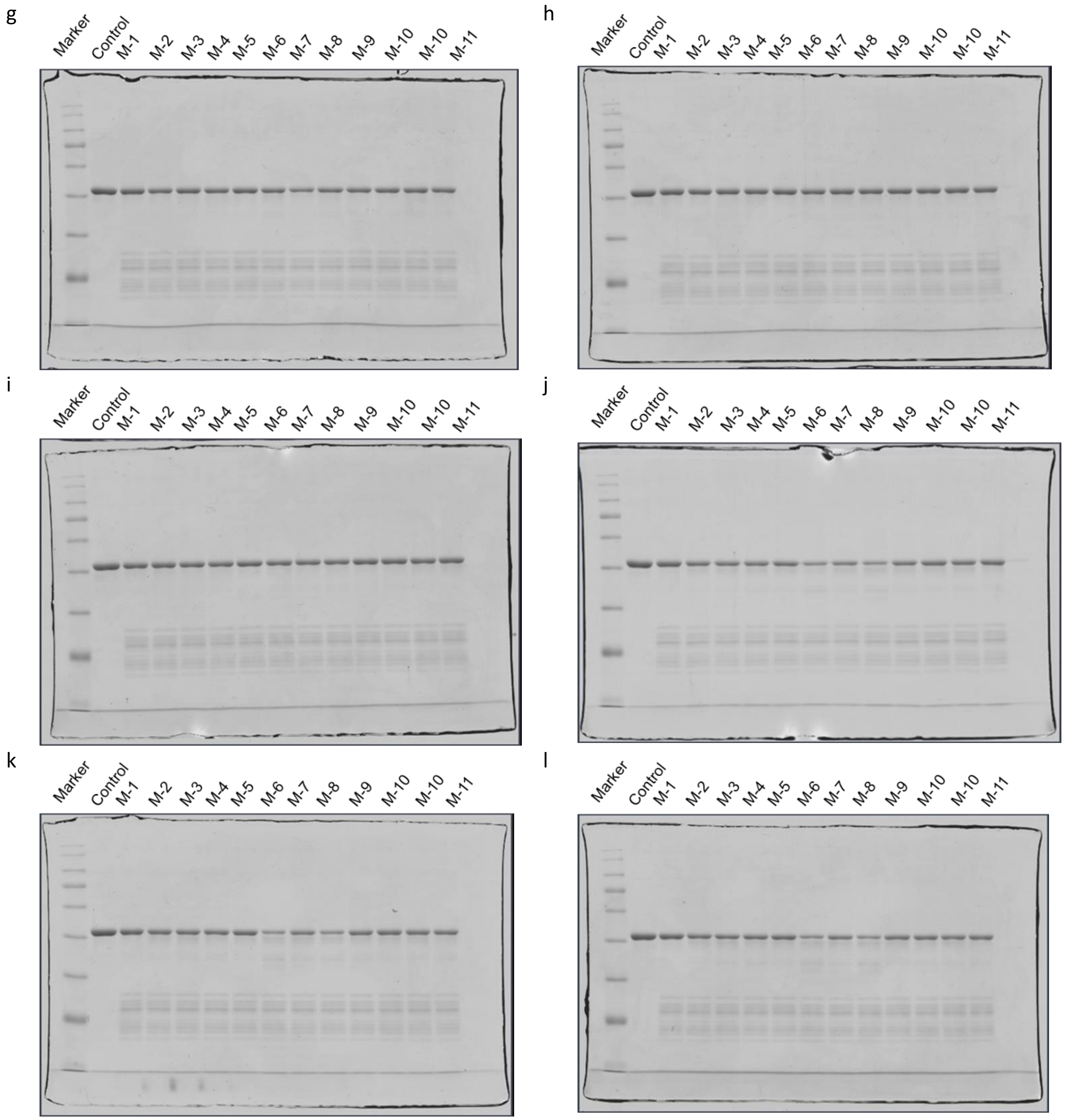

Figure S5. SDS PAGE gels of enolase (a-f), and oxidized enolase (g-l) degradation by the 20S in the presence of the tested peptidomimetics at 1 µM (a-c, g-i) and 10 µM (d-f, j-l) concentration. The experiments were conducted in three replicates. Precision Plus Protein™ Dual Xtra Prestained Protein Standards (Biorad) was used as marker (bands: 2, 5, 10, 15, 20, 25, 37, 50, 75, 100, 150, and 250kDa).

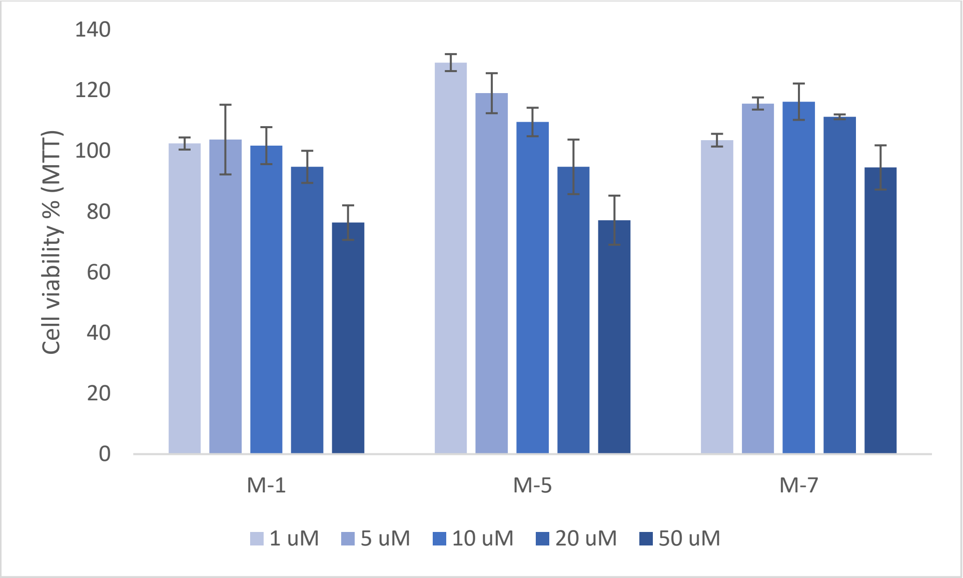

Figure S6. Treatment of HEK293T cells with compounds M-1, M-5, and M-7 (24h) did not significantly affect viability of cells. The results of MTT assay, conducted in at least three independent replicates, are presented as the mean ± SEM.
